# Supplementary material for: The Comparative Safety of Epirubicin and Cyclophosphamide versus Docetaxel and Cyclophosphamide in Lymph Node-Negative, HR-Positive, HER2-Negative Breast Cancer (ELEGANT): A Randomized Trial
Source: Cancers (Basel). 2022 Jun 30;14(13):3221. doi: 10.3390/cancers14133221 (PMC9264845; doi:10.3390/cancers14133221)
Supplement: Supplementary file 1 [file cancers-14-03221-s001.zip › Supplementary file 1.pdf]

CONSORT 2010 checklist of information to include when reporting a randomised trial*

Section/Topic	Item No	Checklist item	Reported on page No
**Title and abstract**			
	1a	Identification as a randomised trial in the title	4
	1b	Structured summary of trial design, methods, results, and conclusions (for specific guidance see CONSORT for abstracts)	4
**Introduction**			
Background and objectives	2a	Scientific background and explanation of rationale	4
	2b	Specific objectives or hypotheses	5
**Methods**			
Trial design	3a	Description of trial design (such as parallel, factorial) including allocation ratio	5
	3b	Important changes to methods after trial commencement (such as eligibility criteria), with reasons	5
Participants	4a	Eligibility criteria for participants	5
	4b	Settings and locations where the data were collected	5
Interventions	5	The interventions for each group with sufficient details to allow replication, including how and when they were actually administered	6
Outcomes	6a	Completely defined pre-specified primary and secondary outcome measures, including how and when they were assessed	6

	6b	Any changes to trial outcomes after the trial commenced, with reasons	6
Sample size	7a	How sample size was determined	6
	7b	When applicable, explanation of any interim analyses and stopping guidelines	6
Randomisation:			6
Sequence generation	8a	Method used to generate the random allocation sequence	
	8b	Type of randomisation; details of any restriction (such as blocking and block size)	7
Allocation concealment mechanism	9	Mechanism used to implement the random allocation sequence (such as sequentially numbered containers), describing any steps taken to conceal the sequence until interventions were assigned	7
Implementation	10	Who generated the random allocation sequence, who enrolled participants, and who assigned participants to interventions	7
Blinding	11a	If done, who was blinded after assignment to interventions (for example, participants, care providers, those assessing outcomes) and how	7
	11b	If relevant, description of the similarity of interventions	7
Statistical methods	12a	Statistical methods used to compare groups for primary and secondary outcomes	7
	12b	Methods for additional analyses, such as subgroup analyses and adjusted analyses	7
**Results**			
Participant flow (a diagram is strongly recommended)	13a	For each group, the numbers of participants who were randomly assigned, received intended treatment, and were analysed for the primary outcome	8
	13b	For each group, losses and exclusions after randomisation, together with reasons	8
Recruitment	14a	Dates defining the periods of recruitment and follow-up	9
	14b	Why the trial ended or was stopped	9
Baseline data	15	A table showing baseline demographic and clinical characteristics for each group	9

Numbers analysed	16	For each group, number of participants (denominator) included in each analysis and whether the analysis was by original assigned groups	11
Outcomes and estimation	17a	For each primary and secondary outcome, results for each group, and the estimated effect size and its precision (such as 95% confidence interval)	12
	17b	For binary outcomes, presentation of both absolute and relative effect sizes is recommended	12
Ancillary analyses	18	Results of any other analyses performed, including subgroup analyses and adjusted analyses, distinguishing pre-specified from exploratory	12
Harms	19	All important harms or unintended effects in each group (for specific guidance see CONSORT for harms)	12
**Discussion**			
Limitations	20	Trial limitations, addressing sources of potential bias, imprecision, and, if relevant, multiplicity of analyses	12
Generalisability	21	Generalisability (external validity, applicability) of the trial findings	12
Interpretation	22	Interpretation consistent with results, balancing benefits and harms, and considering other relevant evidence	13
**Other information**			
Registration	23	Registration number and name of trial registry	13
Protocol	24	Where the full trial protocol can be accessed, if available	13
Funding	25	Sources of funding and other support (such as supply of drugs), role of funders	13

*We strongly recommend reading this statement in conjunction with the CONSORT 2010 Explanation and Elaboration for important clarifications on all the items. If relevant, we also recommend reading CONSORT extensions for cluster randomised trials, non-inferiority and equivalence trials, non-pharmacological treatments, herbal interventions, and pragmatic trials. Additional extensions are forthcoming: for those and for up to date references relevant to this checklist, see www.consort-statement.org.

Item 1a

ELEGANT： Epirubicin and cyclophosphamide versus docetaxel and cyclophosphamide in lymph node negative, HR-positive, HER2-negative breast cancer: A randomized trial

Item 1b

Background

In adjuvant setting, epirubicin and cyclophosphamide (EC), docetaxel and cyclophosphamide (TC) are both optional chemotherapy regimens for lymph node-negative, hormone receptor (HR)-positive, human epidermal receptor 2 (HER2)-negative breast cancer patients. Neutropenia is one of the most common adverse events (AEs) of these regimens. The rate of grade 3-4 neutropenia varies in different studies and safety profile directly comparing EC and TC are lacking.

Method

ELEGANT (NCT02549677) is an observational, prospective, randomized, open-label, non-inferior hematological safety trial. Eligible patients with lymph node-negative HR+/HER2- tumors (1:1) were randomly assigned to received four cycles of EC (90/600 mg/m2) or TC (75/600 mg/m2) every three weeks as adjuvant chemotherapy. The primary endpoint is the incidence of grade 3 or 4 neutropenia defined by National Cancer Institute-Common Terminology Criteria for Adverse Events (NCI-CTCAE) version 4.0 on an intention-to-treat basis. Non-inferiority was defined as an upper 95% CI less than a non-inferiority margin of 15%.

Results

In the intention-to-treat population, 140 and 135 patients were randomized into EC and TC arm, respectively. For the primary endpoint, the rate of grade 3 or 4 neutropenia is 50.71% (95% CI: 42.18%, 59.21%) in EC arm and 48.15% (95% CI: 39.53%, 56.87%) in TC arm (95%CI risk difference: -0.100,0.151), showing non-inferiority of EC arm. For secondary endpoints, the rate of all grade anemia is higher in EC arm (EC 42.86% versus TC 22.96%, $p=0.0007$), and more patients suffer from nausea/vomiting, hair loss and nail changes ($p<0.01$) in EC arm. No statistically different disease-free survival was observed between two arms ($p = 0.13$).

Conclusion

EC is not inferior to TC in the rate of grade 3 or 4 neutropenia, but more other AEs were observed in EC group.

Item 2a

Anthracycline and taxane-based chemotherapy are the cornerstone of adjuvant therapy for early breast cancer. By now, no consensus has been met about the optimal drug combination. In recent years, several trials explored the efficacy of the anthracycline-free regimens, especially for HER2-negative breast cancer patients, which turned out to be a feasible alternative. However, there is no comparison between epirubicin and cyclophosphamide (EC) versus docetaxel and cyclophosphamide (TC) about their safety and efficacy to date. The trial ELEGANT reported a comprehensive safety profile of both regimens with a primary endpoint of grade 3 to 4 neutropenia rate.

Item 2b

Specific objectives and hypothesis

In the current trial we tested the hypothesis of the trial the rate of grade 3 or 4 neutropenia of study group is not inferior to that of control group.

Item 3a

ELEGANT is an observational, prospective, balanced randomized, open-label, non-inferior, hematological safety trial.

Item 3b

None significant changes were made after trial commencement.

Item 4a

Eligible patients were adult female patients younger than 70 years old diagnosed as invasive ER or PR-positive, HER2-negative and lymph node-negative breast cancer with a life expectance of more than 12 months. Baseline blood routine test and other basic tests were done to make sure all participants were in normal hematopoietic, hepatic and renal function. We excluded patients who were allergic, intolerant or poorly compliant to the regimen; previously treated or metastatic breast cancer patients; previously treated with anthracycline or taxane or combined with other malignant tumors (except for controlled cervical carcinoma in situ or skin basal cell carcinoma). Patients who had ⩾1 grade of peripheral neuropathy; on pregnancy or lactation; previously or concurrent enrollment in another trial were also excluded.

Item 4b
The study took place in Comprehensive Breast Health Center, Ruijin Hospital, Shanghai Jiaotong University School of medicine (RJBC) from August 2015 to March 2020. RJBC is one of the largest breast cancer centers in East China with an average malignant breast tumor diagnosis of more than 3000 per year.

Item 5
In ELEGANT, patients were randomized into EC (epirubicin 90mg/m^2, cyclophosphamide 600 mg/m^2) or TC (docetaxel 75mg/m^2, cyclophosphamide 600 mg/m^2) arm for adjuvant chemotherapy. Both were given intravenously. The adjuvant therapy scheme was approved by multidisciplinary-decision team of RJBC. Adjuvant radiotherapy and endocrine therapy regimen were administrated when needed.

Item 6a
The primary endpoint is the incidence of grade 3 or 4 neutropenia in two groups (defined as serum neutrophil granulocyte level <1.0*10^9/L and ⩾0.5*10^9/L for grade 3; <0.5*10^9/L for grade 4). The secondary endpoints were other hematological and non-hematological AEs, 3-year disease free survival and 3-year overall survival. Adverse events were assessed during every cycle of chemotherapy.

Item 6b
No changes of outcome were made after the trial commenced.

Item 7a
To detect non-inferiority of EC, we allowed a difference of up to 15% in the primary outcome. Assuming a neutropenia rate of 40% in EC arm, we need an enrollment of 152 patients per arm for two-sided test to rule out the pre-specified difference in 95% confidence interval (CI) of the noninferiority, allowing for 10% drop-out patients at a two-sided significance level of 0.05 with 80% power.

Item 7b
No interim analysis was made during the trial.

Item 8a

For allocation of participants, a computer-generated list of random number was used.

Item 8b
Participants were equally assigned into two groups following computerized random numbers.

Item 9
Computerized randomization numbers were sequentially sealed in opaque and stapled envelopes. Aluminium foil inside the envelope was used to make the envelope impermeable to intense light. After enrollment, the name of the participant was written on the envelope.

Item 10
Randomization numbers were generated by computer to decide which cytotoxic drug participant should use. Envelope were kept by an investigator not involving the screening and qualification of patients. After obtaining the consent of enrolled patients, randomization began and an envelope was opened by investigator to assign interventions.

Item 11a
ELEGANT is an open-label trial in which patients and physicians were aware of the allocation. However, data analysts were blinded to the allocation.

Item 11b
Not applicable. Allocation was open to patients and investigators.

Item 12a
The primary endpoint is the rate of grade 3 or 4 neutropenia of EC and TC. The secondary endpoints were other hematological and non-hematological AEs, 3-year disease free survival and 3-year overall survival. Chi-square test was applied in the comparison of primary endpoint and other adverse events. Kaplan-Meier method was applied in the survival analyses.

Item 12b

Univariate and multivariate logistic analyses were performed to identify possible predictors of the occurrence of grade 3 or 4 neutropenia.
Item 13a

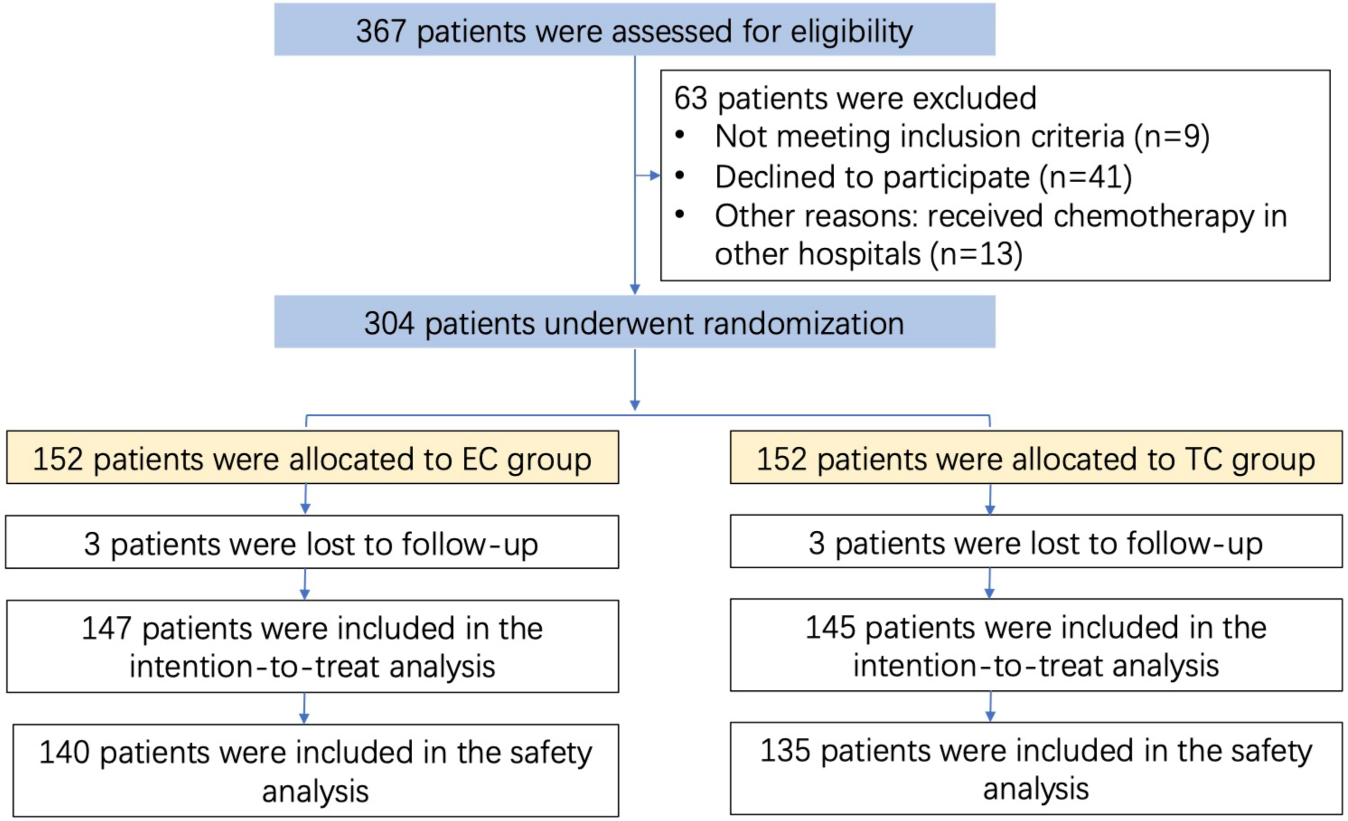

Item 13b

In both groups, 3 patients were lost to follow-up and 2 patients refused to received allocation after randomization. In TC group, 2 patients were excluded from intention-to-treat population because of severe violation of protocol. As a result, there were 147 and 145 participants included in intention-to-treat population. In EC group, 2 patients received prophylactic use of G-CSF and 5 patients had missing record of AEs. In TC group, 1 patient received prophylactic use of G-CSF and 9 patients had missing record of AEs. As a result, there were 140 and 135 participants included in safety population.

Item 14a

All patients were recruited between August 2015 and March 2020 and followed-up by August 2020.

Item 14b

The trial was conducted as scheduled and no early stopping out of plan.

Item 15

	EC n=140(%)	TC n=135(%)	p
Age at diagnosis			0.554
<60	108 (77.14)	99 (73.33)	
⩾60	32 (22.86)	36 (26.67)	
Body Mass Index, kg/m^2			0.463
Underweight (<18.5)	4 (2.86)	7 (5.19)	
Normal Weight (18.5-24.9)	105 (75.00)	101 (74.81)	
Overweight (25-29.9)	27 (19.29)	20 (14.81)	
Obese (⩾30)	4 (2.86)	7 (5.19)	
Number of comorbidities			0.974

0	63 (45.00)	62 (45.93)	
⩾1	77 (55.00)	73 (54.07)	
G-CSF			0.055
Yes	58 (41.43)	40 (29.63)	
No	82 (58.57)	95 (70.37)	
Surgery			0.107
Mastectomy	82 (58.57)	65 (48.15)	
Breast conserving	58 (41.43)	70 (51.85)	
T Stage			0.320
1	105 (75.00)	93 (68.89)	
2	35 (25.00)	42 (31.11)	
PR status			0.219
Negative	25 (17.86)	16 (11.85)	
Positive	115 (82.14)	119 (88.15)	
Ki-67			0.242
<14%	43 (30.71)	32 (23.70)	
⩾14%	97 (69.29)	103 (76.30)	
LVI			0.226
No	130 (92.86)	121 (89.63)	
Yes	6 (4.29)	12 (8.89)	

Unknown	4 (2.86)	2 (1.48)	
Grade			0.507
I	7 (5.00)	5 (3.70)	
II	86 (61.43)	92 (68.15)	
III	32 (22.86)	22 (16.30)	
Unknown	15 (10.71)	16 (11.85)	
Histological type			0.746
Ductal	132 (94.29)	125 (92.59)	
Others	8 (5.71)	10 (7.41)	
21-gene Recurrence Score			0.220
Low risk	4 (2.86)	4 (2.96)	
Median risk	65 (46.43)	79 (58.52)	
High risk	56 (40.00)	43 (31.85)	
Unknown	15 (10.71)	9 (6.67)	
Radiation therapy			0.106
No	80 (57.14)	63 (46.67)	
Yes	60 (42.86)	72 (53.33)	
Endocrine therapy			0.226
SERM-based	65 (46.43)	52 (38.52)	
AI-based	75 (53.57)	83 (61.48)	

Item 16

The primary endpoint and other safety analyses were carried out in the safety population. In EC group, 3 patients were lost to follow-up and 9 patients were protocol violators. 140 patients were included in the safety population for the evaluation of primary endpoint. In TC group, 3 patients were lost to follow-up and 14 patients were protocol violators. 135 patients were included in the safety population.

Item 17a

	EC n=140(%)	TC n=135(%)	Risk difference (95% CI)
Grade 3-4 neutropenia	71 (50.71)	65(48.15)	-0.100, 0.151
All grade neutropenia	131 (93.57)	100 (74.07)	0.103, 0.287

Item 17b

Unapplicable.

Item 18

No subgroup analyses were carried out in the trial.

Item 19

ELEGANT is a trial focusing on adverse events of EC and TC. All possible important harms were recorded in detail in the results. No new AEs were reported in the trial.

Item 20

ELEGANT is an open-label trial with no blinding used among subjects. Apart from the primary endpoint, some other adverse events were reported by patients, which might bring about biases. Non-local patients were permitted to receive allocated therapy in local hospitals according to protocol. Adverse events may be addressed not timely.

Item 21
EC and TC are both recommended and widely used regimens in early HER2-negative breast cancer. This study presented a full review of common adverse events of both regimens especially hematological AEs. The conclusion can be applied in all early HER2-negative breast cancer patients when making treatment decisions and AE prevention and management.

Item 22
Safety endpoint is of great concern in a trial when introducing a new drug or comparing different regimens apart from efficacy endpoints. Our study is the first to compare EC versus TC directly in safety endpoints especially hematological events and showed EC was non-inferior to TC in grade 3 or 4 neutropenia.

Item 23
ELEGANT is registered at ClinicalTrials.gov, number NCT02549677.

Item 24
More details of the trial protocol can be found in the ClinicalTrials.gov and the full text of this article.

Item 25
This trial was financially supported by grants from Shanghai municipal commission of health and family planning (201840323). The funders had no role in study design, data collection and analysis, decision to publish, or preparation of the manuscript.
