# Supplementary figures and images for: The Comparative Safety of Epirubicin and Cyclophosphamide versus Docetaxel and Cyclophosphamide in Lymph Node-Negative, HR-Positive, HER2-Negative Breast Cancer (ELEGANT): A Randomized Trial
Source: Cancers (Basel). 2022 Jun 30;14(13):3221. doi: 10.3390/cancers14133221 (PMC9264845; doi:10.3390/cancers14133221)

## CONSORT 2010 Flow Diagram of trial ELEGANT

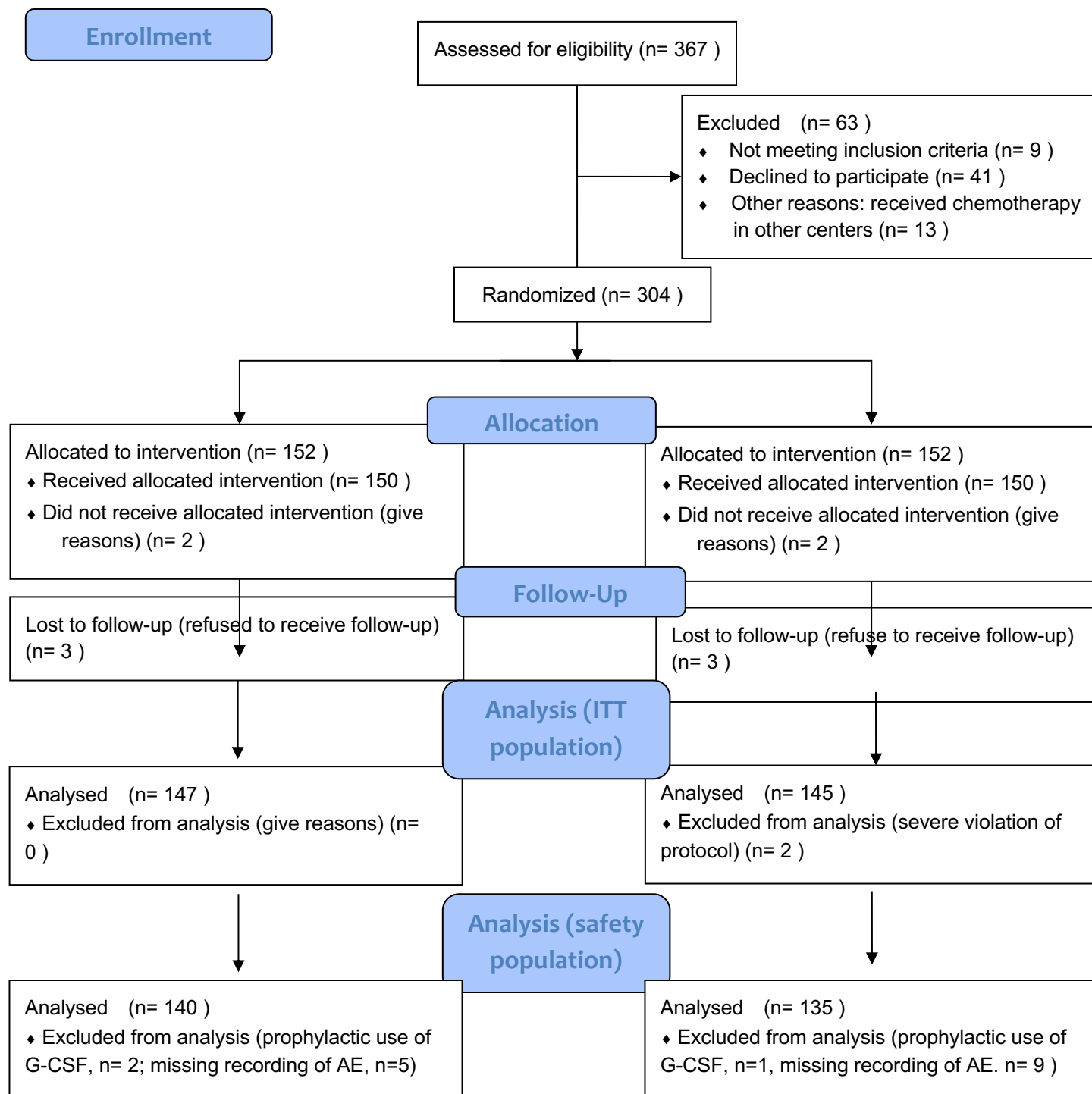

Supplement: Supplementary file 1 [file cancers-14-03221-s001.zip › Supplementary file 2.pdf]
